# Fibronectin Peptide FNIII14 Enhances Progressive Cartilage Degeneration in Osteoarthritis by Inducing Chondrocyte Apoptosis

**DOI:** 10.3390/cimb48060594

**Published:** 2026-06-04

**Authors:** Fumihiro Nishimura, Manabu Sasada, Fumio Fukai, Kyoko Imanaka-Yoshida, Akihiro Sudo, Masahiro Hasegawa

**Affiliations:** 1 Department of Orthopedic Surgery, Graduate School of Medicine, Mie University, 2-174 Edobashi, Mie, Tsu City 514-8507, Japan; fnishimura0715@med.mie-u.ac.jp (F.N.); a-sudou@med.mie-u.ac.jp (A.S.); 2 Faculty of Pharmaceutical Sciences, Tokyo University of Science, 2641 Yamazaki, Chiba, Noda City 278-8510, Japan; msasada@hiroshima-u.ac.jp (M.S.); fukai@rs.noda.tus.ac.jp (F.F.); 3 Clinical Research Center in Hiroshima, Hiroshima University Hospital, 1-2-3 Kasumi, Hiroshima, Minami-Ku 734-8551, Japan; 4 Department of Pathology and Matrix Biology, Graduate School of Medicine, Mie University, 2-174 Edobashi, Mie, Tsu City 514-8507, Japan; imanaka@med.mie-u.ac.jp

**Keywords:** fibronectin, FNIII14, osteoarthritis, chondrocyte, apoptosis, β1 integrin

## Abstract

Osteoarthritis (OA) is highly prevalent worldwide. Fibronectin (FN) has been associated with OA pathology; however, its role remains unexplored. In this study, we hypothesized that FNIII14 induces chondrocyte apoptosis by inactivating β1 integrin and aimed to clarify the role of FNIII14 in OA pathology. Immunohistochemistry, immunofluorescence, flow cytometry, a 3-(4,5-dimethylthiazol-2-yl)-5-(3-carboxymethoxyphenyl)-2-(4-sulfophenyl)-2H-tetrazolium assay, a terminal deoxynucleotidyl transferase deoxyuridine triphosphate nick-end labeling assay, real-time quantitative polymerase chain reaction, and Western blotting were performed using cartilage obtained from patients who underwent total knee arthroplasty. In a mouse model, FNIII14 or phosphate-buffered saline was administered to the knees, and cartilage degeneration and synovitis were evaluated using the Mankin and Synovitis scores, respectively. Statistical significance was determined using the Mann–Whitney U or Kruskal–Wallis test (*p* < 0.05). FNIII14 was detected in highly degenerated OA cartilage, with decreased β1 integrin activity, suppressed cell proliferation, and induced apoptosis in chondrocytes. FNIII14 decreased the gene expression of cartilage-specific markers and anabolic factors and increased inflammatory cytokine gene expression and phosphorylation of extracellular signal-regulated protein kinase-1/2. FNIII14 induced cartilage degeneration in mouse knees with almost no synovitis. Regulation of FNIII14 production and action may play an important role in suppressing cartilage degeneration in human OA.

## 1. Introduction

Globally, more than 500 million people are affected by osteoarthritis (OA), a chronic joint disease with pain, disability, and loss of function being the primary symptoms [1,2]. Most patients with OA have relatively low-grade inflammation [3]. Various factors, including matrix metalloproteinase (MMP)-3, a disintegrin and metalloproteinase with thrombospondin motif (ADAMTS)-4, and interleukin (IL)-1β, are involved in its pathogenesis [4]. No definitive treatment for OA exists; thus, OA is currently treated symptomatically. In 1948, a coagulation protein, cold insoluble globulin [5], was discovered. This protein was later renamed “fibronectin” (FN) when it was found to exhibit cell adhesion activity [6,7]. FN mediates various cellular interactions with the extracellular matrix (ECM), participating in cell adhesion, migration, growth, and differentiation [8]. FN levels are markedly increased in degenerated cartilage in OA [9,10]. However, the effects of FN on OA remain unknown. FN possesses a functional site, FNIII14 (amino acid sequence: TEATITGLEPGTEYTIYVIALC), which opposes cell adhesion [11,12]. This anti-adhesive site is located within the type III module structure in FN but is exposed when FN interacts with MMP-2 [13]. FNIII14 induces a conformation change in β1 integrin, leading to a shift from an active to a resting state [14].

The activation of β1 integrin upregulates type II collagen (COL2) and aggrecan (ACAN) expression, promotes chondrocyte proliferation, and inhibits chondrocyte apoptosis [15]. We hypothesized that FNIII14 reverses these effects by inactivating β1 integrin in chondrocytes. Therefore, we investigated the involvement of FNIII14 in OA pathology by interrogating the effects of FNIII14 on chondrocytes in vitro and explored whether the intra-articular injection of FNIII14 can induce OA in mice.

## 2. Materials and Methods

### 2.1. Isolation and Culture of Chondrocytes

We isolated chondrocytes from the articular cartilage of the tibial plateaus and femoral condyles of patients with advanced OA who underwent total knee arthroplasty between May 2021 and January 2023. Patients were excluded if (1) the amount of cartilage required for the experiment could not be harvested owing to significant cartilage damage, (2) they had rheumatoid arthritis or osteonecrosis, or (3) they underwent revision surgery. Chondrocytes from cartilage specimens were isolated under sterile conditions and cultured as described previously [16]. The study protocol involving human tissues was approved by the ethics committee of the Clinical Research Support Center, Mie University (approval number: H2020-235; approved on 25 November 2020), and informed consent was obtained from all participants.

### 2.2. Immunohistochemistry

To immediately fix specimens obtained from patients, 0.1% phosphate-buffered saline (PBS; pH 7.4), including 4% paraformaldehyde, was used at 22 °C for 7 days. Specimens were embedded in paraffin after being decalcified with treated K-CX (Falma, Tokyo, Japan) and cut into 5 µm thick sections. Safranin-O (Saf-O) or hematoxylin and eosin (H&E) was used to stain the sections. Saf-O staining was used to evaluate the proteoglycan content within cartilage tissue, whereas H&E staining was used to assess general tissue morphology and cellular structure. We immunohistochemically analyzed FNIII14 expression. To retrieve antigens from sections, 0.04% Proteinase K (Sigma-Aldrich, St. Louis, MO, USA) was used at 22 °C for 10 min. Sections with 0.5 µg/mL FNIII14 antibody were incubated at 4 °C overnight and washed. Subsequently, the sections were incubated with peroxidase-conjugated anti-rabbit immunoglobulin (Ig)G fragment antigen-binding region (1:100 dilution; DAKO, Glostrup, Denmark) at 37 °C for 1 h. Immune reactions were visualized using 0.15 mg/mL of 3,3′-diaminobenzidine tetrahydrochloride (Dojindo Lab., Kumamoto, Japan)/hydrogen peroxidase (Fujifilm, Wako Pure Chemical Co., Osaka, Japan) solution.

### 2.3. Immunofluorescence

After the cells reached 80–90% confluence on the culture slides, chondrocytes were maintained under serum-free conditions and cultured with or without 0.1% bovine serum albumin (BSA), including 10 µg/mL FNIII14 for 24 h. After washing, the cells were fixed in 2% paraformaldehyde before being incubated with anti-cluster of differentiation (CD)29 mouse IgG monoclonal antibody (β1 integrin, AG89; 1:100 dilution; human; D050-3, monoclonal antibody, MBL, Tokyo, Japan) overnight. Subsequently, the cells were incubated with anti-mouse-IgG Alexa Fluor 488 antibody for 4 h in the dark. The stained cells were visualized using BX53, DP74, a U-LGPS light source, and CellSens Standard 1.18 (build16686; Olympus Corp, Tokyo, Japan).

### 2.4. Flow Cytometry

Flow cytometry (FCM) was performed to analyze β1 integrin activity and chondrocyte apoptosis. After chondrocytes reached 80–90% confluence on 10 cm dishes, they were maintained under serum-free conditions and either left untreated or treated with 0.1% BSA, including 10 µg/mL FNIII14 for 24 h. For β1 integrin activation analysis, chondrocytes were incubated with stained anti-CD29 mouse IgG (MBL). For apoptosis analysis, chondrocytes were incubated with 0 or 10 µg/mL FNIII14 for 0, 6, and 24 h, followed by propidium iodide and annexin V–fluorescein isothiocyanate double staining (Beckman Coulter, Miami, FL, USA), and were analyzed using a BD Accuri C6 Flow Cytometer (Becton, Dickinson and Company, Franklin Lakes, NJ, USA).

### 2.5. Measurement of Cell Viability

To examine the effects of FNIII14 on chondrocyte proliferation, we performed proliferation assays (n = 7) using the CellTiter 96^®^ AQueous Non-Radioactive Cell Proliferation Assay kit (Promega, Madison, WI, USA). Chondrocytes (5.0 × 10^3^ cells/well) were plated and cultured in 96-well plates with Dulbecco’s modified Eagle medium/F12 containing 10% BSA for 24 h; treated with 0, 1, 5, 10, or 20 µg/mL FNIII14; and cultured for an additional 7 days before initiating the proliferation assay. Optical density at 490 nm 2 h after treatment on days 0 and 7 was measured using infinite^®^ F200 PRO (TECAN Group Ltd., Männedorf, Zürich, Switzerland). Optical density values were normalized using a blank control.

### 2.6. Terminal Deoxynucleotidyl Transferase Deoxyuridine Triphosphate Nick-End Labeling Assay

After the cells reached 80–90% confluence on the culture slides, chondrocytes under serum-free conditions were treated with 0, 1, or 10 µg/mL FNIII14 containing 0.1% BSA for 6, 12, and 24 h (n = 5). After washing, the cells were fixed in 2% paraformaldehyde. The ApopTag^®^ Peroxidase In Situ Apoptosis Detection kit (Invitrogen, Waltham, MA, USA) was used for terminal deoxynucleotidyl transferase deoxyuridine triphosphate nick-end labeling (TUNEL) staining. All groups were processed simultaneously under identical staining and 3,3′-diaminobenzidine development conditions to minimize technical variability. The stained cells were visualized using a light microscope. Images of TUNEL-stained specimens were taken 24 h after treatment at five random locations and at 200× magnification; total and positively stained cells were counted. We defined “% total” as the ratio of the number of positively stained cells to the total cell number and compared the values.

### 2.7. RNA Extraction and Complementary DNA Synthesis and Real-Time Quantitative Polymerase Chain Reaction

After the cells reached 80–90% confluence in six-well plates, chondrocytes maintained under serum-free conditions were treated with 0, 1, or 10 µg/mL FNIII14 containing 0.1% BSA for 24 h. Total RNA was isolated from ten samples using the RNeasy Mini Kit (QIAGEN, Venlo, The Netherlands) and reverse-transcribed as previously described [16]. The sex-determining region of Y chromosome-box transcription factor 9 (SOX9) was tested in all samples, and other factors were tested in at least six samples. We evaluated the expression of the following chondrocyte-specific markers (SOX-9, n = 10; COL2 alpha 1 chain [COL2A1], n = 7; ACAN, n = 8), inflammatory cytokines (tumor necrosis factor [TNF]-α, n = 8; IL-1B, n = 6; IL-6, n = 6), anabolic factors (basic fibroblast growth factor 2 [FGF-2], n = 9; tissue inhibitor of metalloproteinase [TIMP]3, n = 9; transforming growth factor β [TGFB], n = 7), and catabolic factors (MMP-2, n = 7; MMP-3, n = 6; MMP-9, n = 7; MMP-13, n = 6; ADAMTS4, n = 6; ADAMTS5, n = 6). Primer–probe pairs for the TaqMan gene expression assay were used to detect the expression of *SOX9* (Hs00165814_m1), *COL2A1* (Hs00264051_m1), *ACAN* (Hs00153936_m1), *TNFA* (Hs00174128_m1), *IL-1B* (Hs01555410_m1), *IL-6* (Hs00174131_m1), *FGF2* (Hs00266645_m1), *TIMP3* (Hs00165949_m1), *TGFB* (Hs00998133_m1), *MMP2* (Hs01548727_m1), *MMP3* (Hs00968305_m1), *MMP9* (Hs00957562_m1), *MMP13* (Hs00233992_m1), *ADAMTS4* (Hs00192708_m1), *ADAMTS5* (Hs00199841_m1), and *GAPDH* (Hs99999905_m1; Thermo Fisher Scientific, Waltham, MA, USA). The ABI Prism 7000 Sequence Detector System (Applied Biosystems, Foster City, CA, USA) was used for complementary DNA (cDNA) quantification. TaqMan Universal PCR Master Mix (Roche Diagnostics, Penzberg, Germany) was used to determine target gene expression. The conditions of thermal cycling were as follows: 50 °C for 2 min, 95 °C for 10 min, 40 cycles at 95 °C for 15 s, and 60 °C for 1 min. Cytokine mRNA levels were normalized to those of *GAPDH*, which were compared between the FNIII14-treated and -untreated cells.

### 2.8. Western Blotting

After the cells reached 80–90% confluence in six-well plates, chondrocytes were maintained under serum-free conditions and left untreated or treated with 1 or 10 µg/mL FNIII14 containing 0.1% BSA for 24 h. Next, radioimmunoprecipitation buffer (Millipore-Upstate, Temecula, CA, USA) containing a protease inhibitor cocktail (0.5 mM phenylmethylsulphonyl fluoride and 0.2 mM trisodium orthovanadate) was used for cell lysis. Sodium dodecyl sulphate (10%)–polyacrylamide gel electrophoresis was used for protein separation, and an equal quantity of protein was analyzed for each sample. The separated proteins were transferred onto nitrocellulose membranes, and the expression of the following proteins was examined: protein kinase B (Akt), n = 7; phosphorylated (p)-Akt, n = 7; extracellular signal-regulated kinase (ERK) 1/2, n = 6; p-ERK 1/2, n = 6; and SOX-9, n = 7. The following primary antibodies were used at the recommended dilutions: anti-β-actin (goat monoclonal, ab8229, Abcam, Cambridge, UK; 1:500); Akt (rabbit polyclonal, #9272), anti-p-Akt (rabbit polyclonal, Ser473, #9271), anti-ERK 1/2 (rabbit polyclonal, p44/42 mitogen-activated protein kinase [MAPK], #9102; all Cell Signaling Technology, Beverly, MA, USA; 1:1000); anti-p-ERK 1/2 (rabbit monoclonal, Thr202/Tyr204, #4370, Cell Signaling Technology; 1:2000); and anti-SOX9 antibody (goat polyclonal, sc-17341, Santa Cruz Biotechnology, Dallas, TX, USA; 1:100). Anti-mouse Ig secondary antibodies conjugated with horseradish peroxidase (HRP) to the corresponding primary antibody (anti-goat Ig/HRP and anti-rabbit Ig/HRP; DAKO) were used. Immunoreactive bands were detected by chemiluminescence using Amersham™ ECL™ prime (GE Healthcare Technologies Inc., Chicago, IL, USA). β-actin was used as the loading control. LAS-4000 mini (Fujifilm) was used to detect each protein band. The relative density of each protein band was analyzed using ImageJ open-source software (version 1.53t; US National Institutes of Health, Bethesda, MD, USA, http://imagej.nih.gov/ij/ [accessed on 28 September 2022]).

### 2.9. Reagents and Antibodies

FNIII14 peptide was synthesized using a solid-phase strategy combined with *tert*-butyloxycarbonyl and fluorenyl methoxycarbonyl protecting group chemistry. By adding cysteine to the carboxyl terminal of each peptide, its activity was increased owing to dimerization, and binding to silica gel beads was promoted. We purified the synthesized FNIII14 peptide using reversed-phase high-performance liquid chromatography and then characterized it using mass spectrometry [17].

The function-blocking antibody against peptide FNIII14 was prepared by Tokyo University of Science (Chiba, Japan). We immunized rabbits with a synthesized peptide (CLEPGTEYTIYVIALK), including the active sequence bound to thyroglobulin, applied the IgG fraction in rabbit sera to Sepharose beads bound to the synthesized peptide immunogen, and used the IgG eluate as the anti-FNIII14 antibody. The conjugate of ovalbumin and peptide FNIII14 was prepared with maleimide-activated ovalbumin.

### 2.10. Animals

Twenty-four 8-week-old male BALB/c mice (Japan SLC, Inc., Hamamatsu, Shizuoka, Japan) weighing approximately 22 g were maintained in line with guidelines approved by the Animal Experiment and Care Committee of Mie University Faculty of Medicine. The study protocol was approved by the relevant institutional review board (approval number 2022-19; approval date 6 March 2023).

### 2.11. Intra-Articular Injection of FNIII14 to BALB/c Mice

Intra-muscular injection of sodium pentobarbital (0.05 mg/g body weight) was used to anaesthetize the mice. Through the trans-patella tendon approach, 10 or 100 µg/mL FNIII14 (10 µL) was injected into the knee joints (10 µg/mL = group C, n = 6; 100 µg/mL = group D, n = 6). The control group was designed to consider only the effect of the surgical approach and intra-articular injection, and thus received a PBS injection (10 µL; group B, n = 6). Untreated mice were designated as the non-operation (normal) group (group A, n = 6). We randomly divided the mice into these groups, from A to D. Mice could walk freely without splinting after surgery. The mice were maintained under standard conditions (n = 6/cage, temperature: 24–25 °C, 12 h light–dark cycle) in the laboratory’s animal house and were provided water and standard mouse chow ad libitum. Cervical dislocation was used to sacrifice the mice at 12 weeks after FNIII14 injection. We then microscopically analyzed the knees of mice to observe the effect of FNIII14.

### 2.12. Evaluation

Paraformaldehyde (4%) was used to fix all samples at 22 °C for 5 days, and 10% EDTA was used for decalcification. Next, the samples were embedded in paraffin after dehydration and coronally sliced at 5 µm thickness. Saf-O and H&E were used to stain the sections. A blinded single investigator scored all specimens. Synovitis and cartilage degeneration were evaluated on the basis of the synovitis score and the Mankin score, respectively [18,19]. Synovial membranes were scored based on the following three features: synovial lining cell layer, stroma cell density, and inflammatory infiltrate, with the ranking of alterations ranging from none (0), slight (1), moderate (2), to strong (3). Notably, grade 9 denoted the most severe/high-grade synovitis. The Mankin score provides a combined score for cartilage structure, as follows: normal (0), surface irregularities (1), pannus and surface irregularities (2), clefts to transitional zone (3), clefts to radial zone (4), clefts to calcified zone (5), and complete disorganization (6). For cellular abnormalities, the scores were as follows: normal (0), diffuse hypercellularity (1), cloning (2), hypocellularity (3). For matrix staining, the scores were as follows: normal (0), slight reduction (1), moderate reduction (2), severe reduction (3), and no dye noted (4). Lastly, the scores for tidemark integrity were as follows: intact (0) and crossed by blood vessels (1). Higher scores show increased OA severity. We compared the scores obtained from the medial tibial plateaus for all groups (A–D).

### 2.13. Statistical Analyses

Statistical significance was determined using the Kruskal–Wallis or Mann–Whitney U test in the Statistical Package for Social Sciences version 27 software (IBM Corp., Armonk, NY, USA); significance was set at *p* < 0.05. The data are expressed as the mean ± standard deviation.

## 3. Results

### 3.1. FNIII14 Is Highly Expressed in Degenerated Human OA Cartilage

**Figure 1:**
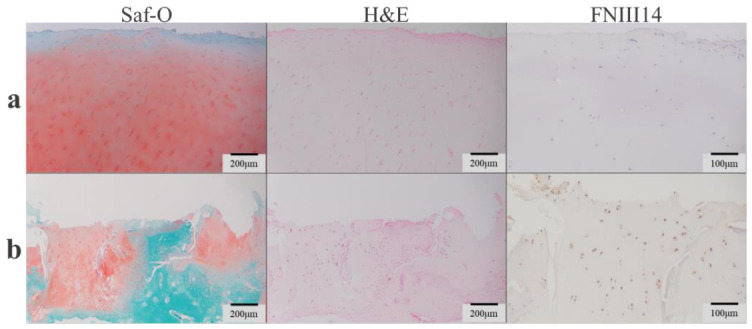
Histological and immunohistochemical images of human cartilage subjected to Saf-O, HE, and FNIII14 staining. Representative staining images showing the (**a**) non-degenerated osteoarthritis (OA) cartilage and (**b**) degenerated OA cartilage of the same joint of a patient. FNIII14 expression was higher in degenerated OA cartilage than in non-degenerated OA cartilage. Saf-O, H&E scale bars = 200 µm. FNIII14 scale bars = 100 µm. Saf-O, safranin-O; H&E, hematoxylin and eosin.

Normal Saf-O staining was observed for non-degenerated OA cartilage (Figure 1a), whereas degenerated OA cartilage showed an irregular surface and weak Saf-O staining (Figure 1b). Immunohistochemistry showed that FNIII14 was highly expressed in degenerated OA cartilage compared with non-degenerated OA cartilage (Figure 1a,b).

### 3.2. β1 Integrin Activity Decreases in FNIII14-Treated Chondrocytes

**Figure 2:**
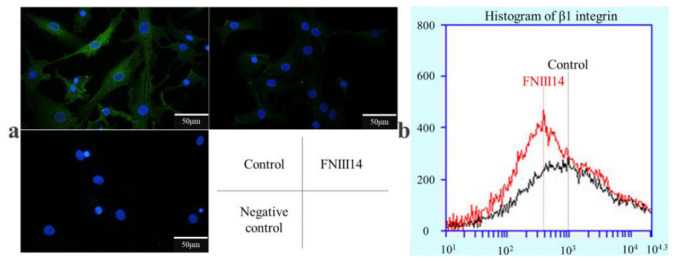
Representative immunofluorescence images (**a**) and histogram (**b**) of β1 integrin. FNIII14-treated cells showed weaker staining and lower β1 integrin activity than untreated cells. Scale bars = 50 µm.

Untreated cells showed strong β1 integrin staining, whereas FNIII14-treated cells showed weak β1 integrin staining (Figure 2a). FCM showed that β1 integrin activity decreased in FNIII14-treated cells compared with that in untreated cells (Figure 2b).

### 3.3. Inhibition of Chondrocyte Proliferation by FNIII14

**Figure 3:**
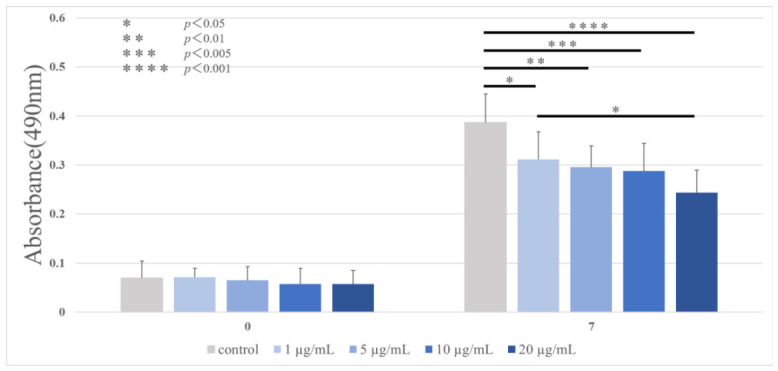
Cell proliferation assay results. Compared with the untreated cell count, the FNIII14-treated cell count significantly decreased on day 7. Cells treated with 20 µg/mL FNIII14 had a significantly lower count than cells treated with 1 µg/mL FNIII14. n = 7, Kruskal–Wallis test.

Compared with the number of untreated cells, that of FNIII14-treated cells significantly decreased on day 7 (1 µg/mL, *p* = 0.032; 5 µg/mL, *p* = 0.009; 10 µg/mL, *p* = 0.004; and 20 µg/mL, *p* < 0.001; Figure 3). FNIII14-treated cells exhibited a significantly lower count than cells treated with 1 µg/mL FNIII14 (*p* = 0.029).

### 3.4. Induction of Chondrocyte Apoptosis with FNIII14

**Figure 4:**
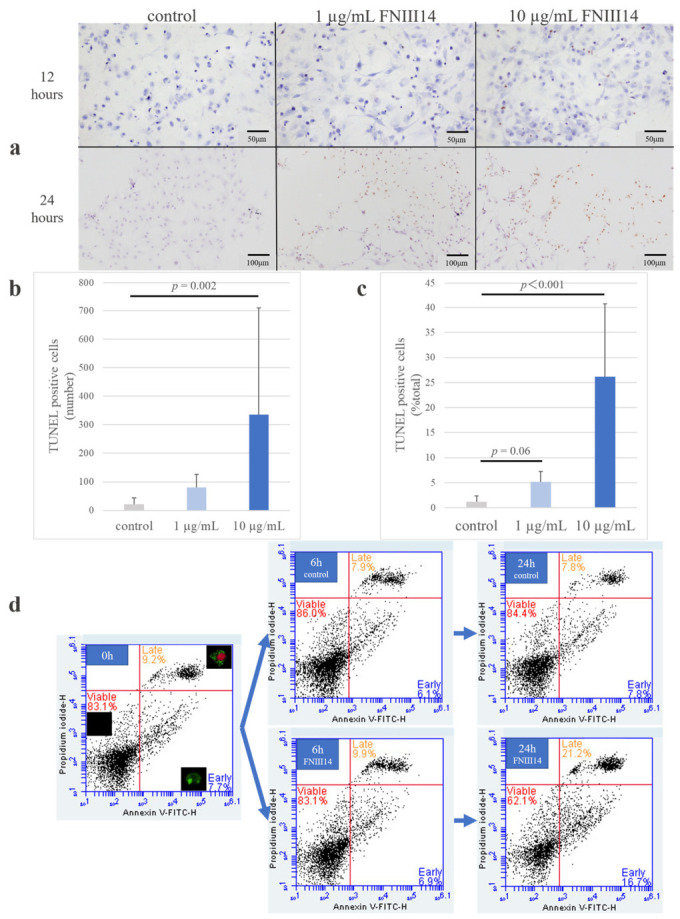
TUNEL assay and flow cytometry with annexin V. (**a**) Representative staining images of TUNEL-stained specimens 12 or 24 h after FNIII14 treatment. (**b**) Number of TUNEL-positive cells (n = 5, Kruskal–Wallis test). (**c**) Ratio of TUNEL-positive cells to the total number of cells (% total) 24 h after FNIII14 treatment (n = 5, Kruskal–Wallis test). The number and ratio of TUNEL-positive cells increased significantly in the 10 µg/mL FNIII14-supplemented group and tended to increase even in the 1 µg/mL FNIII14-supplemented group. (**d**) Quantification of apoptotic cell population. Depending on the fluorescence intensity of annexin V (x-axis) and propidium iodide (y-axis), the cell populations were classified as double-negative (viable) cells, annexin V-positive only (early apoptotic) cells, and double-positive (late apoptotic) cells. In the figure, 12 h scale bars = 50 µm, 24 h scale bars = 100 µm. TUNEL, terminal deoxynucleotidyl transferase deoxyuridine triphosphate nick-end labeling; FITC, fluorescein isothiocyanate.

No TUNEL-positive chondrocytes were observed at any concentration following a 6 h incubation with FNIII14; however, TUNEL-positive chondrocytes were observed after incubation for 12 h with 10 µg/mL FNIII14 and 24 h with 1 and 10 µg/mL FNIII14 (Figure 4a). The number of TUNEL-positive cells increased significantly in the 10 µg/mL FNIII14-supplemented group (*p* = 0.002; Figure 4b). The percentage total increased significantly in the 10 µg/mL FNIII14-supplemented group (*p* < 0.001) and tended to increase in the 1 µg/mL FNIII14-supplemented group (*p* = 0.06; Figure 4c). After treatment for 6 h, no differences were observed in cell distribution between FNIII14-treated and control groups. However, with 24 h treatment, the early apoptosis rate (EAR; only annexin V-positive zone) and late apoptosis rate (LAR; both annexin V- and propidium iodide-positive zones) increased in the FNIII14-treated group (EAR = 16.7%, LAR = 21.2%) compared with the control group (EAR = 7.8%, LAR = 7.8%; Figure 4d).

### 3.5. Gene Expression Changes in FNIII14-Treated Chondrocytes

**Figure 5:**
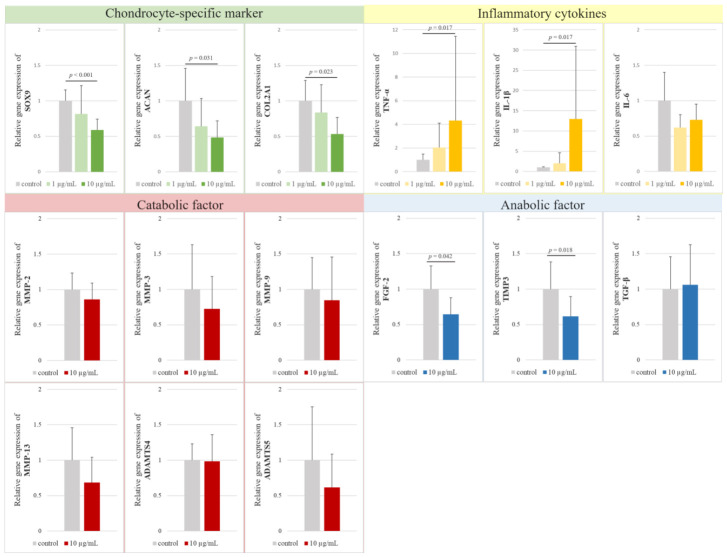
Gene expression analysis (*SOX-9*, n = 10; *ACAN*, n = 8; *COL2A1*, n = 7; *TNF-α*, n = 8; *IL-1B*, n = 6; *IL-6*, n = 6; *MMP-2*, n = 7; *MMP-3*, n = 6; *MMP-9*, n = 7; *MMP-13*, n = 6; *ADAMTS4*, n = 6; *ADAMTS5*, n = 6; *FGF-2*, n = 9; *TIMP3*, n = 9; *TGFB*, n = 7). Chondrocyte-specific markers and inflammatory cytokines were compared using the Kruskal–Wallis test; catabolic factors and anabolic factors were compared using the Mann–Whitney U test. Treatment with 10 µg/mL FNIII14 downregulated the expression of chondrocyte-specific markers (*SOX9*, *ACAN*, and *COL2A1*) and anabolic factors (basic *FGF2* and *TIMP3*) but upregulated the expression of inflammatory cytokines (*TNF-α* and *IL-1β*). SOX9, sex-determining region of the Y chromosome-box transcription factor 9; ACAN, aggrecan; COL2A1, collagen type II alpha 1 chain; TNF, tumor necrosis factor; IL, interleukin; MMP, matrix metalloproteinase; FGF, fibroblast growth factor; TIMP, tissue inhibitor of metalloproteinase; TGF, transforming growth factor; ADAMTS, a disintegrin and metalloproteinase with thrombospondin motif.

Compared with that in the control group, the mRNA expression of chondrocyte-specific markers (*SOX9*, *p* < 0.001; *COL2A1*, *p* = 0.023; and *ACAN*, *p* = 0.031) and anabolic factors (*FGF2*, *p* = 0.042 and *TIMP3*, *p* = 0.018) was downregulated in the group treated with 10 µg/mL FNIII14; however, this treatment upregulated the mRNA expression of inflammatory cytokines (*TNFA*, *p* = 0.017 and *IL-1B*, *p* = 0.017; Figure 5). Notably, the mRNA expression of catabolic factors (*MMP-2*, *p* = 0.38; *MMP-3*, *p* = 0.58; *MMP-9*, *p* = 0.31; *MMP-13*, *p* = 0.24; *ADAMTS4*, *p* = 0.81; *ADAMTS5*, *p* = 0.31; *IL-6*, *p* = 0.40; and *TGFB*, *p* = 0.90) did not differ significantly. Furthermore, treatment with 1 µg/mL FNIII14 did not alter the mRNA expression of chondrocyte-specific markers (*SOX9*, *p* = 0.12; *COL2A1*, *p* = 0.83; and *ACAN*, *p* = 0.21) or inflammatory cytokines (*TNFA*, *p* = 0.67; *IL-1B*, *p* = 1.0; and *IL-6*, *p* = 0.13).

### 3.6. Upregulation of ERK1/2 Phosphorylation in FNIII14-Treated Chondrocytes

**Figure 6:**
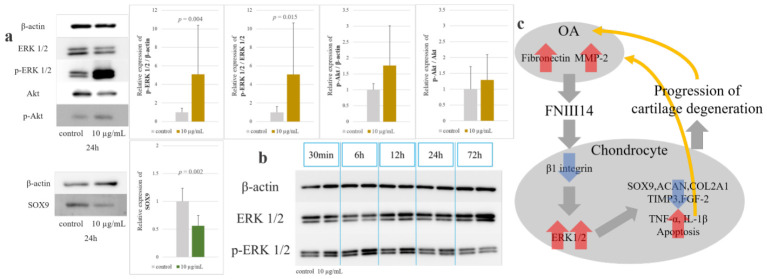
(**a**) Western blot analysis showing protein expression quantified using ImageJ (ERK1/2, n = 6; p-ERK1/2, n = 6; Akt, n = 7; p-Akt, n = 7; and SOX9, n = 7, Mann–Whitney U test). The phosphorylation of ERK1/2 was significantly upregulated, but that of SOX9 was significantly downregulated after FNIII14 (10 µg/mL) treatment compared with the control group. (**b**) Time course of ERK1/2 phosphorylation. ERK1/2 was highly phosphorylated with 10 µg/mL FNIII14 treatment compared with controls until 24 h after administration; however, ERK1/2 reached the same level as that of the control after 72 h. (**c**) Schema of FNIII14 production and progression of cartilage degeneration. FNIII14 is produced when fibronectin (FN) is processed by MMP-2. FNIII14 reduces β1 integrin activity and dysregulates ERK1/2. Moreover, FNIII14 decreases the expression of cartilage-specific markers and anabolic factors, increases the expression of genes encoding inflammatory cytokines, and induces chondrocyte apoptosis. Through these collective effects, FNIII14 induces cartilage degeneration progression. FN and MMP-2 are produced as cartilage degeneration progresses. FNIII14 production increases as MMP-2 generation increases due to IL-1β, further accelerating cartilage degeneration. ERK, extracellular signal-regulated kinase; Akt, protein kinase B; p, phosphorylated; OA, osteoarthritis; SOX9, sex-determining region of the Y chromosome-box transcription factor 9; ACAN, aggrecan; COL2A1, collagen type II alpha 1 chain; TNF, tumor necrosis factor; IL, interleukin; MMP, matrix metalloproteinase; FGF, fibroblast growth factor; TIMP, tissue inhibitor of metalloproteinase.

Compared with the control group, treatment with 10 µg/mL FNIII14 significantly increased ERK1/2 phosphorylation (p-ERK1/2/β-actin, *p* = 0.004; p-ERK1/2/ERK1/2, *p* = 0.015) and significantly decreased SOX9 (SOX9/β-actin, *p* = 0.002) expression, but Akt phosphorylation showed no significant difference (p-Akt/β-actin, *p* = 0.48; p-Akt/Akt, *p* = 0.43; Figure 6a). ERK1/2 phosphorylation increased after 30 min and up to 24 h of treatment with 10 µg/mL FNIII14 compared with that in controls. However, phosphorylated ERK1/2 levels were comparable to those observed in controls after 72 h (Figure 6b). Chondrocytes treated with FNIII14 enhanced ERK1/2 phosphorylation (Figure 6c).

### 3.7. Degenerated Cartilage and Minimal Synovitis After Intra-Articular Injection of FNIII14

**Figure 7:**
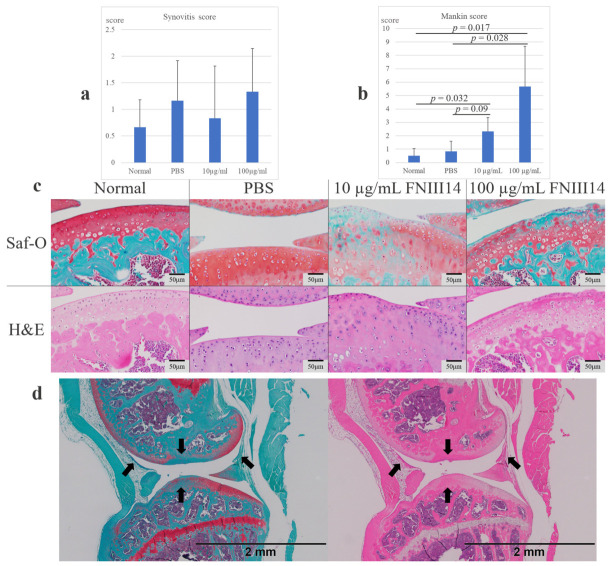
(**a**) Synovitis score (n = 6 in each group, Kruskal–Wallis test). No significant differences in synovitis scores were observed among the following groups: Normal (group A), PBS (group B), 10 µg/mL FNIII14 (group C), and 100 µg/mL FNIII14 (group D). (**b**) Mankin score (n = 6 in each group, Kruskal–Wallis test). The cartilage of mice in group C was significantly more degenerated than that of mice in group A. Although not significant, the cartilage of mice in group C was more degenerated than that of mice in group B. The cartilage of mice in group D was significantly more degenerated than that of mice in groups A and B. (**c**) Representative staining images of tissue sections of the tibial cartilage from mice of groups A–D. Weaker Saf-O staining was noted in groups C and D than in groups A and B. In group C, the cartilage surface was slightly distorted, whereas in group D, marked surface irregularity and severe structural disruption were consistently observed. Group D showed sparse chondrocyte distribution and reduced cellularity within the degenerated cartilage. (**d**) Representative staining images of tissue sections of the knee treated with 100 µg/mL FNIII14. Significant cartilage degeneration was observed in tibial and femoral cartilage, with almost no synovitis. Panel (**c**) scale bars = 50 µm. Panel (**d**) scale bars = 2 µm. PBS, phosphate-buffered saline; Saf-O, safranin-O; H&E, hematoxylin and eosin.

The synovitis scores among groups A–D at 12 weeks after intra-articular injection did not show any significant differences (*p* = 0.43; Figure 7a). However, Mankin scores suggested that the knees of the mice in groups C and D were significantly degenerated compared with those of mice in group A (group C, *p* = 0.032; group D, *p* = 0.017). Although not significant for group C, the knees of the mice in group D were more degenerated than those of mice in group B (group C, *p* = 0.09; group D, *p* = 0.028; Figure 7b). Mankin scores among groups A and B (*p* = 0.85) and groups C and D (*p* = 0.26) did not differ significantly. Weak Saf-O staining was observed in groups C and D compared with that in groups A and B. The cartilage surface was slightly distorted in group C, whereas marked surface irregularity and severe structural disruption were consistently observed in group D. The degenerated cartilage in group D showed sparse chondrocyte distribution with reduced cellularity (Figure 7c). Considerable degeneration was observed in the tibial and femoral cartilages, with almost no synovitis in the knees of group D mice (Figure 7d).

## 4. Discussion

The average concentration of FN in synovial fluids from patients with OA was over three-fold higher than that in healthy donors, consistent with increased FN levels observed in OA cartilage [20,21]. Additionally, MMP-2 expression was markedly upregulated in the cartilage of patients with OA during the later stages of cartilage degeneration [22,23]. Therefore, FNIII14 expression may be elevated in degenerated OA cartilage. Herein, FNIII14 expression was observed in the cartilage of patients with OA, and FNIII14 production markedly increased in the articular cartilage that underwent more advanced degeneration, suggesting that FNIII14 may directly contribute to cartilage degeneration in patients with OA.

In vitro IF and FCM results showed that FNIII14 reduced β1 integrin activity in chondrocytes. This study suggests that FNIII14 induced an effect opposite to that of β1 integrin activation in chondrocytes, consistent with previous findings [14,24,25]. OA is increasingly recognized as a mechanobiological disorder characterized by complex interactions among ECM degradation, abnormal mechanotransduction, and altered joint biomechanics [26]. Changes in the mechanical properties of cartilage and mechanobiological signaling pathways contribute substantially to progressive joint degeneration and functional impairment. Under physiological conditions, articular cartilage homeostasis is maintained through the ability of chondrocytes to appropriately sense and respond to mechanical stimuli. In contrast, disruption of cartilage homeostasis leads to an imbalance between anabolic and catabolic signaling pathways, resulting in ECM degradation and chondrocyte apoptosis [27]. Among the molecular regulators involved in these processes, integrin receptors have emerged as critical mediators of cartilage homeostasis and OA progression through their roles in mechanotransduction, ECM signaling, cytoskeletal organization, and inflammatory responses [26,28]. β1 integrins play essential roles in mechanotransduction, in which mechanical stimuli are converted into intracellular biochemical responses [29,30,31]. Functional inactivation of β1 integrins by FNIII14 may impair the ability of chondrocytes to appropriately sense and adapt to physiological mechanical loading. Such disruption of mechanotransduction may result in reduced anabolic maintenance of the ECM and increased susceptibility to mechanically induced degeneration. In late-stage OA, the cartilage becomes hypocellular owing to chondrocyte death [32]. Chondrocyte apoptosis is considered a critical event in OA progression because the loss of viable chondrocytes compromises ECM maintenance and tissue repair capacity. Thus, FNIII14, which induces chondrocyte apoptosis and suppresses chondrocyte proliferation, may play an important role in OA progression by contributing not only to cellular loss but also to the progressive deterioration of cartilage structural integrity. TNFA and IL-1B regulate the degeneration of the articular cartilage matrix, and IL-1B also induces MMP-2 expression in human chondrocytes via a prostaglandin E2-dependent mechanism [33,34]. Therefore, increased FNIII14 expression caused by MMP-2-mediated FN processing may exhibit a positive-feedback effect (Figure 6c). This finding indicates that FNIII14 may contribute to a self-amplifying degenerative cascade once its local concentration exceeds a certain level. However, the threshold conditions required for sustained cartilage degeneration remain unclear and should be investigated in future studies. Furthermore, FNIII14 decreased the expression of genes encoding cartilage-specific markers, such as COL2 and ACAN, which are major components of the cartilage, and SOX9, an essential transcription factor in the cartilage [35]. Although FNIII14 did not markedly alter the expression of genes encoding catabolic factors, it significantly decreased the expression of the anabolic factor genes FGF2 and TIMP3, suggesting that FNIII14 induces a relatively catabolic-dominant state by disrupting cartilage homeostasis and creating an imbalance between anabolic and catabolic signaling pathways.

FNIII14 reportedly decreases Akt phosphorylation in NIH3T3 cells [36]; however, in the present study, ERK1/2 phosphorylation was promoted, whereas Akt phosphorylation was not. FN fragments are reportedly involved in the MAPK pathway in chondrocytes [37], and the signaling pathways activated may differ depending on the cell type. Dysregulation of cellular processes governed by ERK proteins contributes to human diseases [38], including cancers [39,40] and neuro-cardio-facial-cutaneous syndromes [41,42]. Additionally, alterations in intracellular signaling pathways, particularly the MAPK pathway, play a prominent role in chondrocyte dysfunction, which contributes to OA pathogenesis and progression [43]. Both ERK inhibition and activation can induce chondrocyte apoptosis [44,45,46,47]; therefore, the effects of the ERK signaling pathway on chondrocytes are multifaceted and require further evaluation. The findings of the present study showed that ERK1/2 activation induced chondrocyte apoptosis, suggesting that ERK dysregulation negatively impacts chondrocytes. Recent studies have demonstrated that FN fragments are not merely passive degradation products of the ECM, but rather biologically active matrikines that regulate integrin-mediated signaling and contribute to OA progression [26]. Our findings suggest that FNIII14 may function as a matrikine-like peptide that contributes to cartilage degeneration through integrin-associated signaling pathways. Collectively, these observations suggest that FN fragments actively modulate integrin signaling and promote catabolic processes in osteoarthritic cartilage.

We observed that a single FNIII14 injection into mouse knee joints induced cartilage degeneration after 12 weeks. Furthermore, marginal or no synovitis was noted following FNIII14 injection. In an earlier study, mice showed mild cartilage degeneration 12 weeks after intra-articular FNIII14 administration compared with a mouse model of monoiodoacetic acid-induced arthritis [48]. However, unlike monoiodoacetic acid, FNIII14 exists in vivo and may induce more physiological cartilage degeneration. The time-period results of Western blotting and in vivo experiments provided further evidence that FNIII14 may be involved in accelerating cartilage degeneration.

This study has some limitations. We conducted in vitro experiments using cartilage tissues from patients who underwent total knee arthroplasty. Thus, chondrocytes were isolated from deteriorated and depleted cartilage tissues, thereby limiting the sample size. Moreover, owing to ethical constraints associated with obtaining cartilage tissue and chondrocytes from healthy donors, a comparison of the effects of FNIII14 between normal human chondrocytes and those derived from patients with OA could not be performed. In addition, we did not evaluate the biomechanical properties of the cartilage, such as stiffness, elasticity, or frictional characteristics. Quantitative biomechanical analyses, including micro-indentation or tribological testing, may further clarify the relationship between FNIII14-induced molecular alterations and structural failure of cartilage tissue [49,50]. Furthermore, behavioral analyses such as gait assessment or locomotor activity monitoring were not performed in the mouse experiments. Such evaluations may help clarify the functional consequences of FNIII14-induced cartilage degeneration. Moreover, although the sample size in the mouse experiments was comparable to that in previous exploratory biological studies, it may not have been sufficient to establish biomechanical reliability or fully evaluate inter-sample variability in cartilage degeneration. Nevertheless, histological alterations, including reduced Saf-O staining and surface structural disruption, were reproducibly observed in FNIII14-treated specimens, supporting the consistency of the observed degenerative changes. Finally, although we confirmed that FNIII14 was present in the human articular cartilage, its levels could not be quantified.

## 5. Conclusions

FNIII14 was found to induce cartilage degeneration with minimal synovitis. FNIII14 inactivated β1 integrin in chondrocytes, suppressed *ACAN* and *COL2* expression, suppressed chondrocyte proliferation, and induced apoptosis. Thus, our findings confirm our hypotheses, suggesting that regulating FNIII14 production and activity may aid in suppressing cartilage degeneration in patients with OA, ultimately contributing to the development of therapeutic drugs that mitigate OA severity. Verifying that FNIII14 promotes cartilage degeneration in fresh human chondrocytes differentiated from human mesenchymal stem cells and that anti-FNIII14 antibodies suppress the progression of this degeneration may address the limitations of this study and provide further insights into OA pathology.

## Data Availability

The original contributions presented in this study are included in the article. Further inquiries can be directed to the corresponding author.

## Abbreviations

The following abbreviations are used in this manuscript:

ACAN	aggrecan
ADAMTS	a disintegrin and metalloproteinase with thrombospondin motif
Akt	protein kinase B
BSA	bovine serum albumin
CD	cluster of differentiation
cDNA	complementary DNA
COL2	type II collagen
COL2A1	COL2 alpha 1 chain
EAR	early apoptosis rate
ECM	extracellular matrix
ERK	extracellular signal-regulated kinase
FCM	flow cytometry
FGF	fibroblast growth factor
FITC	fluorescein isothiocyanate
FN	fibronectin
H&E	hematoxylin and eosin
HRP	horseradish peroxidase
IF	immunofluorescence
Ig	immunoglobulin
IL	interleukin
LAR	late apoptosis rate
MAPK	mitogen-activated protein kinase
MMP	matrix metalloproteinase
OA	osteoarthritis
p	phosphorylated
PBS	phosphate-buffered saline
Saf-O	safranin-O
SOX9	sex-determining region of the Y chromosome-box transcription factor-9
TGFB	transforming growth factor β
TIMP	tissue inhibitor of metalloproteinase
TNFA	tumor necrosis factor-α
TUNEL	terminal deoxynucleotidyl transferase deoxyuridine triphosphate nick-end labeling
